# Differences at surgery between patients with bicuspid and tricuspid aortic valves

**DOI:** 10.1007/s12471-018-1214-1

**Published:** 2018-12-13

**Authors:** L. Cozijnsen, H. J. van der Zaag-Loonen, M. A. Cozijnsen, R. L. Braam, R. H. Heijmen, B. J. Bouma, B. J. M. Mulder

**Affiliations:** 1 Department of Cardiology, Gelre Hospital, Apeldoorn, The Netherlands; 2 Department of Epidemiology, Gelre Hospital, Apeldoorn, The Netherlands; 3 Department of Paediatric Gastroenterology and Hepatology, Erasmus Medical Centre, Rotterdam, The Netherlands; 4 Department of Cardiothoracic Surgery, Nieuwegein, The Netherlands; 5 Department of Cardiology, Amsterdam University Medical Centre, location AMC, Amsterdam, The Netherlands

**Keywords:** Aortic valve surgery, Native valve anatomy, Bicuspid aortic valve, Tricuspid aortic valve, Aortic stenosis, Cardiovascular risk factors

## Abstract

**Aim:**

To determine differences in surgical procedures and clinical characteristics at the time of surgery between native bicuspid aortic valves (BAV) and tricuspid aortic valves (TAV) in patients being followed up after aortic valve surgery (AVS).

**Methods:**

In this retrospective cohort study in a non-academic hospital, we identified patients who had a surgeon’s report of the number of native valve cusps and were still being followed up. We selected patients with BAV and TAV, and used multivariable regression analyses to identify associations between BAV-TAV and pre-specified clinical characteristics.

**Results:**

Of 439 patients, 140 had BAV (32%) and 299 TAV (68%). BAV patients were younger at the time of surgery (mean age 58.6 ± 13 years) than TAV patients (69.1 ± 12 years, *p* < 0.001) and were more often male (64% vs 53%; *p* = 0.029). Cardiovascular risk factors were less prevalent in BAV than in TAV patients at the time of surgery (hypertension (31% vs 55%), hypercholesterolaemia (29% vs 58%) and diabetes (7% vs 16%); all *p* < 0.005). Concomitant coronary artery bypass grafting (CABG) was performed less often in BAV than in TAV patients (14% vs 39%, *p* < 0.001), even when adjusted for confounders (adjusted odds ratio (adj.OR) 0.45; 95% CI: 0.25–0.83). In contrast, surgery of the proximal aorta was performed more often (31% vs 11%, respectively, *p* < 0.001; adj.OR 2.3; 95% CI: 1.3–4.0).

**Conclusions:**

Whereas mechanical stress is the supposed major driver of valvulopathy towards AVS in BAV, prevalent cardiovascular risk factors are a suspected driver towards the requirement for AVS and concomitant CABG in TAV, an observation based on surgical determination of the number of valve cusps.

## What’s new?

- In comparison with patients with bicuspid aortic valves, patients with tricuspid aortic valves (TAV) more often had hypercholesterolaemia at the time of surgery.
- TAV patients more often underwent concomitant coronary artery bypass grafting.
- Hypercholesterolaemia is a probable major driver in TAV disease.
- The role of statins in TAV disease deserves further study.

## Introduction

Bicuspid aortic valve (BAV) is the most common congenital heart defect with an estimated prevalence of 0.5–2% in the general population and a male predominance of approximately 3:1 [1]. Several studies have reported familial clustering of BAV; however, determining the genetics of BAV is complex [1]. BAV may lead to valvular dysfunction, mostly calcific aortic stenosis (AS) and is frequently associated with ascending aortic dilatation, termed ‘BAV aortopathy’.

The development of AS in BAV is related to the abnormal valve geometry with mechanical or tensile stress as the supposed major driver of the progressive character of stenosis. Histopathological studies have shown that in the development of AS, bicuspid and tricuspid aortic valves (TAV) share features of inflammation, neovascularisation, lipid deposition and calcification [2–4]. It is suggested that the aetiopathogenetic process of bicuspid and tricuspid valvulopathy differs mainly in the earlier onset of disease in BAV related to the increased mechanical stress on the cusps.

The Cardiovascular Health Study and the CANHEART Aortic Stenosis Study demonstrated that the well-known cardiovascular risk factors age, male gender, hypertension, smoking, dyslipidaemia and diabetes were associated with the development and progression of AS [5, 6]. Additionally, greater attainment of ideal cardiovascular health in midlife to later life was associated with a lower prevalence of AS in late life [7]. These results are often extrapolated to BAV stenosis. However, all three studies excluded patients with BAV. Studies that distinguished between BAV and TAV stenosis in relation to cardiovascular risk factors were rather small [8, 9]. This lack of distinguishing between BAV and TAV is likely related to the difficulty in reliably diagnosing BAV by echocardiography in the case of AS. Indeed, in the ASTRONOMER trial, valve morphology was uncertain in almost 20% of patients [10].

Some studies have pointed at differences between BAV and TAV [11, 12]. Analysis of possible differences in the process of valvulopathy is important for possible preventive treatment adjusted to the aetiopathogenetic process. A previous study demonstrated that knowledge of native valve anatomy is essential for appropriate follow-up after aortic valve replacement (AVR) [13]. The current study aimed to assess differences between BAV and TAV patients being followed up after aortic valve surgery (AVS) based on surgical determination of the number of cusps and focussed on differences in pre-operative clinical profile and surgical procedures in order to detect possible targets for preventive strategies.

## Methods

### Study population and definitions

**Fig. 1:**
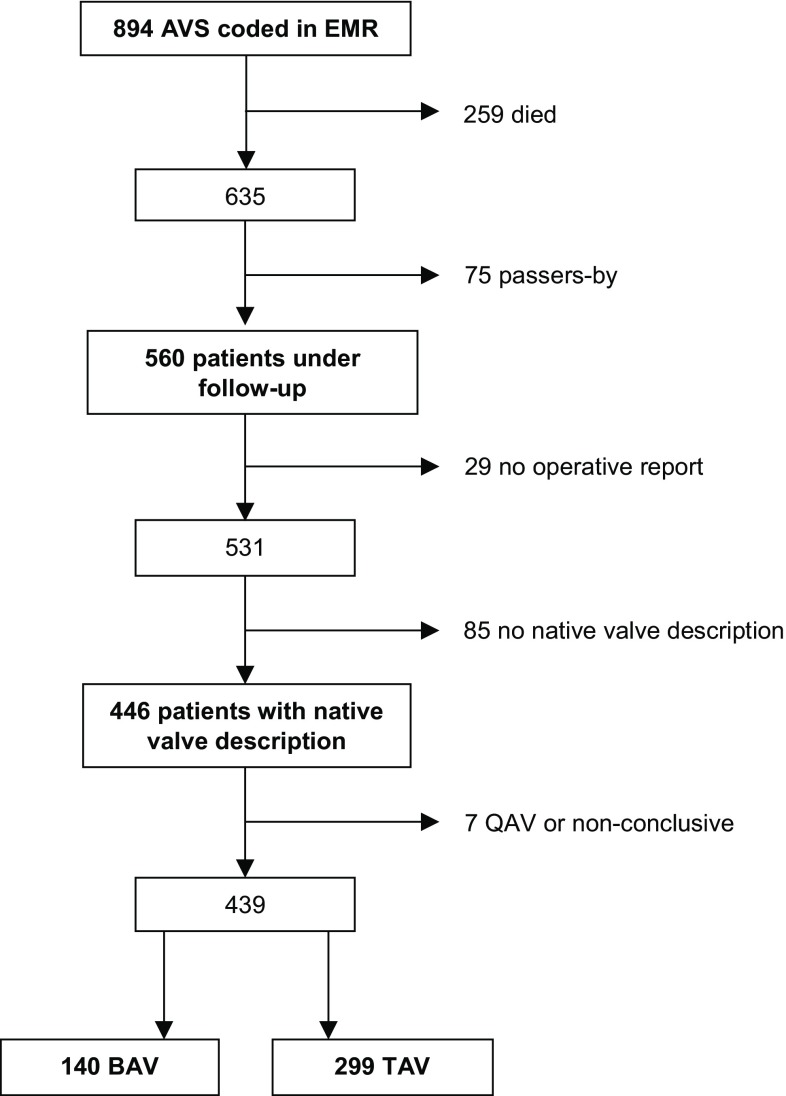
Patient selection. *AVS* aortic valve surgery, *BAV* bicuspid aortic valve, *EMR* electronic medical record, *QAV* quadricuspid aortic valve, *TAV* tricuspid aortic valve

In 2012 all patients with BAV or TAV disease being followed up after AVS were identified from the electronic medical record (EMR) system in Gelre Hospital, Apeldoorn, a non-academic teaching hospital without a cardiothoracic surgery unit (Fig. 1). AVS was defined as replacement or valvuloplasty of the aortic valve or replacement of the aortic root. Transcatheter aortic valve implantations were excluded as the aortic valve is not visualised. Clinical data were retrieved from the EMR; details of surgery and native valve anatomy were documented from the operative report. Surgical assessment was used to determine the number of cusps. The reliability of this method was discussed in a previous publication referring to many studies that used the intra-operative description by the surgeon for determination of valve anatomy [13].

Cardiovascular risk factors were assessed at the time of surgery. Hypertension was defined as being on antihypertensive treatment and diabetes as being on antidiabetic medication. Hypercholesterolaemia was defined as a serum total cholesterol >7.0 mmol/l or being on cholesterol-lowering medication.

Proximal aortic surgery was defined as comprising all surgical procedures involving the aortic root and/or the ascending aorta [i. e. aortic root replacement, Bentall procedure, supracoronary ascendens replacement (SCAR) and ascending aorta reefing/patch enlargement]. A Bentall procedure was indicated if there was an indication for AVR and a pre-operative aortic root or ascending aorta diameter of ≥4.5 cm during AVR, or a thin-walled aorta at inspection at the discretion of the surgeon. SCAR was usually performed in cases of a dilated ascending aorta but a non-dilated aortic root. Replacement of the aortic arch was usually performed if the dilatation included the distal ascending aorta (≥4.5 cm or 4.0 cm and thin-walled).

### Statistical analysis

Normally distributed continuous variables were tested with the Student *t*-test, not normally distributed variables with the Mann-Whitney test. Dichotomous variables were tested with the chi-squared statistic or Fisher’s exact statistic in the case of small numbers. Logistic multivariable regression analyses were used to assess the association between the BAV/TAV status and the aortic valve procedure and additional surgery. Linear regression analysis was used to assess the association with the prosthesis diameter. Adjustment was performed for potential confounders, i. e. variables with a univariate association with BAV/TAV status indicated by a *p*-value <0.10, with a maximum of one confounder per 10 cases. A significance level of <0.05 was considered significant, providing 95% confidence intervals. We used IBM Statistical Package for the Social Sciences (SPSS) 24.

The institutional review board judged that this study fell outside the scope of the Dutch law of medical-scientific research with humans (WMO), and therefore patient consent was not required.

## Results

Of 560 patients being followed up after AVS, 439 had a surgeon’s report determining them as having either a BAV (*n* = 140; 32%) or a TAV (*n* = 299; 68%) (Fig. 1; [13]). BAV patients were younger than TAV patients at the time of surgery (mean age 58.6 ± 13.4 years vs 69.1 ± 11.7 years, *p* < 0.001) and were more often male (64% vs 53%, *p* = 0.029) (Tab. 1).

**Table 1:** Clinical characteristics of bicuspid (*BAV*) and tricuspid aortic valve (*TAV*) patients

*n* = 439	BAV (*n* = 140)	TAV (*n* = 299)	*p*-value
Mean age at surgery, years (SD)	58.6 (13.4)	69.1 (11.7)	<0.001
Age range, years (min–max)	18–86	22–89	
Male, *n* (%)	90 (64)	159 (53)	0.029
Year of surgery (IQR)	2005 (1999–2009)	2007 (2003–2010)	<0.001
Years since surgery, median (IQR)	7.8 (3.8–13.5)	5.3 (2.4–9.5)	<0.001
Former LVOTO/CoA operation, *n* (%)	4 (2.9)^a^	2 (0.7)^b^	0.91
Rheumatic fever, *n* (%)	1 (0.7)	4 (1.3)	0.57
Endocarditis (active or old), *n* (%)	8 (5.7)	14 (4.7)	0.64
Hypertension, *n* (%)	44 (31)	165 (55)	<0.001
Hypercholesterolaemia, *n* (%)	40 (29)	172 (58)	<0.001
Diabetes, *n* (%)	10 (7.1)	49 (16)	0.005

*CoA* aortic coarctation, *IQR* interquartile range, *LVOTO* left ventricular outflow tract obstruction, *SD* standard deviation

^a^Aortic coarctation (*n* = 2)

^b^Aortic coarctation (*n* = 1)

The surgical procedures were performed between 1971 and 2012, and most patients (359/439; 82%) were operated on in two main referral centres.

The operations of BAV patients had been performed a median of 7.8 years previously (interquartile range (IQR): 3.8–13.5), those of TAV patients more recently (median 5.3 years previously, IQR 2.4–9.5, *p* < 0.001). The prevalence of hypertension (31%), hypercholesterolaemia (29%) and diabetes (7%) was lower in BAV than TAV patients (55, 58 and 16%, respectively; all *p* values <0.005) (Tab. 1). After adjustment for age at surgery, gender and years since surgery, the prevalence of hypertension and diabetes was no longer significantly different, but the prevalence of hypercholesterolaemia remained different between BAV and TAV patients (*p* = 0.001).

The underlying valve dysfunctions indicating surgery are demonstrated in Tab. 2. The indication for surgery was mainly AS (67%), followed by aortic insufficiency (16%) or combined AS and insufficiency (7.6%). The remaining patients underwent surgery for aortic aneurysm or dissection, endocarditis, or a concomitant indication such as coronary artery disease (CAD) or mitral valve disease. Concomitant AVS was performed in 21 patients (4.8%), less often in BAV than in TAV patients (1.4% vs 6.4%, *p* = 0.03).

**Table 2:** Indications for surgery

*N* = 437^a^	Total	BAV (*n* = 139)	TAV (*n* = 298)	*p*-value
Aortic stenosis, *n* (%)	293 (67)	97 (70)	196 (66)	0.45
Aortic insufficiency, *n* (%)	69 (16)	18 (13)	51 (17)	0.32
Combined aortic stenosis and insufficiency, *n* (%)	33 (7.6)	14 (10)	19 (6.4)	0.18
Concomitant indication^b^, *n* (%)	21 (4.8)	2 (1.4)	19 (6.4)	0.03
Aneurysm, dissection, *n* (%)	13 (3.0)	5 (3.6)	8 (2.7)	0.56
Endocarditis, *n* (%)	8 (1.8)	3 (2.2) ^c^	5 (1.7) ^c^	0.71

*BAV* bicuspid aortic valve, *TAV* tricuspid aortic valve

^a^Two patients without data on indication

^b^Concomitant indications: coronary artery disease (*n* = 10), mitral valve disease (*n* = 9), myxoma (*n* = 1), hypertrophic cardiomyopathy (*n* = 1)

^c^Including two cases with aortic insufficiency

Tab. 3 shows the surgical procedures performed. BAV patients more often underwent surgery including the aortic root and ascending aorta; aortic valve procedures were less often limited to isolated valve replacement (104/140, 74%) than in TAV patients (275/299, 92%; adjusted odds ratio (OR): 0.49; 95% CI: 0.26–0.92).

**Table 3:** Aortic valve procedures and additional procedures

*N* = 439	BAV (*n* = 140)	TAV (*n* = 299)	Crude OR (95% CI)	Adjusted OR^a^ (95% CI)
*I. Aortic valve procedures*				
Isolated valve prosthesis^b^, *n* (%)	104 (74)	275 (92)	0.25 (0.14–0.44)	0.49 (0.26–0.92)
– Bentall procedure, *n* (%)	28 (20)*	19 (6.4)	3.68 (1.98–6.87)	‡
– Aortic root replacement^c^, *n* (%)	2 (1.4)	2 (0.7)	2.15 (0.30–15.4)	–
– Other aortic valve procedures^d^, *n* (%)	6 (4.3)	3 (1.0)	4.42 (1.09–17.9)	‡
*II. Additional procedures*				
CABG, *n* (%)	20 (14)	118 (39)	0.26 (0.15–0.43)	0.45 (0.25–0.83)
SCAR,* n* (%)	8 (5.7)	5 (1.7)	3.56 (1.14–11.1)	‡
Arch replacement, *n* (%)	11 (7.9)	8 (2.7)	3.10 (1.21–7.89)	‡
Other aortic surgery^e^, *n* (%)	7 (5.0)*	8 (2.7)	1.63 (0.55–4.79)	–
Mitral and/or tricuspid surgery, *n* (%)	11 (7.9)	41 (14)	0.54 (0.27–1.79)	–
Other cardiac surgery^f^, *n* (%)	8 (5.7)	29 (9.7)	0.56 (0.25–1.27)	–
*III. ‘Proximal aortic surgery’*	44 (31)	34 (11)	3.57 (2.16–5.92)	2.3 (1.3–4.0)

*BAV* bicuspid aortic valve, *CABG* coronary artery bypass grafting, *CI* confidence interval, *OR* odds ratio, *SCAR* supracoronary ascending replacement, *TAV* tricuspid aortic valve

*P < 0.001; ‡ numbers too small for multivariable analysis

^a^Adjusted for age at surgery, gender, years since surgery, hypertension, hypercholesterolemia and diabetes

^b^Mechanical or biological

^c^Two cases of endocarditis and two cases of elective homograft by choice

^d^Homograft (*n* = 2), autograft (*n* = 4), valvuloplasty (*n* = 3)

^e^Ascending aorta reefing/patch enlargement

^f^Congenital correction (*n* = 8), rhythm surgery (*n* = 16), Morrow procedure (*n* = 3), myxoma, and various patch-plasties

A mechanical valve prosthesis was implanted in 41% of patients (178/436) and a biological prosthesis in 56% (248/436; 3/439 had valvuloplasty). Only 2.3% (10 patients) received a homograft or an autograft. A mechanical valve prosthesis was more often used in BAV than in TAV patients (58% vs 32%), the difference disappearing after adjustment for confounders.

The diameter of the implanted prosthesis was on average larger in BAV than in TAV patients (25 ± 2.3 mm vs 24 ± 2.3 mm, mean difference adjusted for confounders: 0.45; 95% CI: 0.02–0.88).

**Fig. 2:**
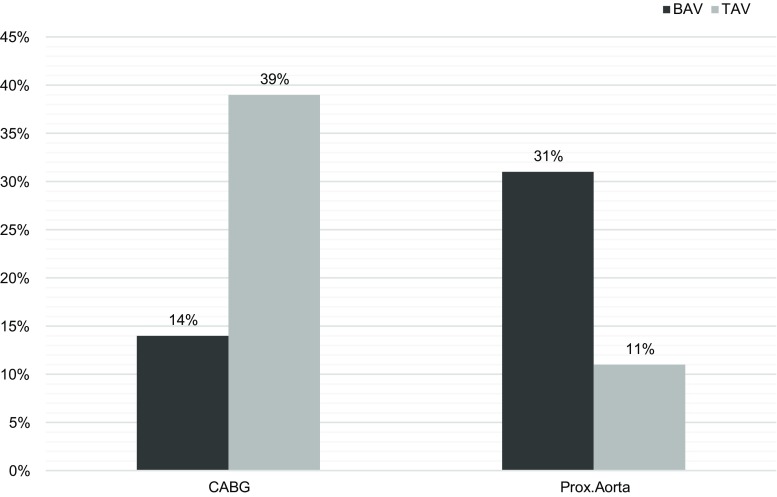
Concomitant coronary artery bypass grafting (*CABG*) and proximal aortic (*Prox.Aorta*) surgery. Bicuspid (*BAV*, *n* = 140) versus tricuspid aortic valves (*TAV*, *n* = 299). Adjusted odds ratio for CABG 0.45 (95% CI: 0.25–0.83) and for Prox.Aorta 2.3 (95% CI: 1.3–4.0), both adjusted for age at surgery, gender, years since surgery and years since surgery, hypertension, hypercholesterolaemia and diabetes

Additional procedures are shown in Tab. 3. Concomitant coronary artery bypass grafting (CABG) was performed less often in BAV than in TAV patients (14% vs 39%, adjusted OR: 0.45; 95% CI: 0.25–0.83). In contrast, concomitant ‘proximal aortic surgery’ was performed in BAV patients more often (31% vs 11%; adjusted OR: 2.3; 95% CI: 1.3–4.0) (Fig. 2).

## Discussion

At the time of AVS and compared to TAV patients, BAV patients: (1) had hypercholesterolaemia less often (29% vs 58%); (2) had larger prostheses implanted (25 mm vs 24 mm); (3) underwent concomitant proximal aortic surgery more often (31% vs 11%); (4) underwent concomitant CABG less often (14% vs 39%).

Determination of the number of native valve cusps was based on surgical assessment. In calcified, stenotic aortic valves, echocardiography is often not reliable for diagnosis, and surgical assessment is viewed to be the gold standard [14] and was used as such in many previous studies [13, 15–17].

### Cardiovascular risk factors, CABG and AVR

In our study, in many patients the presence of CAD and aortic valvular disease (AVD) required surgical intervention simultaneously, driven by cardiovascular risk factors, but only in TAV patients, as BAV patients less often underwent concomitant CABG and had less prevalent cardiovascular risk factors. A recent systematic review reported that at the time of AVR, in all 16 studies the need for concomitant CABG was lower for BAV than for TAV. A meta-regression analysis showed that this was explained by younger age and lower prevalence of diabetes in BAV compared to TAV patients [18]. In contrast, our study demonstrated that native valve anatomy determined the need for CABG in addition to age and hypercholesterolaemia. Davies et al. [11] and Boudoulas et al. [12] observed that the need for concomitant CABG was lower in BAV than in TAV patients of every age group. As in our study, the difference in the need for CABG was associated not only with age but also with native valve anatomy.

On the other hand, the difference in cardiovascular risk factors at the time of AVR also points to possible differences in the development of AVD between BAV and TAV. A study by Huntley et al. found that after age-matching, patients with TAV compared with BAV stenosis had more cardiovascular risk factors, including hypertension, hyperlipidaemia and diabetes [19]. Similarly, in our study patients with TAV had more cardiovascular risk factors than BAV patients, with hypertension and diabetes mellitus being associated with age, but hypercholesterolaemia remained more prevalent in TAV after multivariate analysis.

These results point towards hypercholesterolaemia as a possible major driver in tricuspid AVD, which is not or less the case in bicuspid AVD. In contrast to eight (of the nine) observational studies, the five randomised controlled trials (RCTs) with statin therapy failed to show any benefit regarding the progression of AS. However, it might be questioned whether the negative results of these RCTs exclude the possibility that hypercholesterolaemia might be a major driver in tricuspid AVD [20]. The two largest studies among these RCTs, the SEAS and the ASTRONOMER, comprised 5.3 and 49% patients with BAV [10, 21]. We are not aware of sub-studies reporting on the effect of statins in TAV patients only. Furthermore, it must be noted that the SEAS and the ASTRONOMER studies excluded patients with a clinical indication for cholesterol lowering, such as CAD and diabetes which diminished the prevalence of cardiovascular risk factors in the study and hence also diminished the amount of ‘typical tricuspid AS’ patients. We agree with the conclusion of a recent systematic review that the exclusion of patients with the greatest risk of atherosclerosis and the relative brief follow-up of the RCTs likely reduced the possibility that statins would produce a therapeutic effect [20]. A study of tricuspid AS patients which takes this into account might be considered, as the recent review article also suggested [20].

### Prosthesis diameter and aortic surgery

Valve prosthesis diameters were on average larger in BAV than in TAV, which was also reported by Huntley et al. [19]. The mean difference in diameter between BAV and TAV in their study was 0.8 mm, while this was 1 mm in ours, and after adjustment 0.5 mm. A difference between the studies must be noted: Huntley et al. compared BAV patients with age-adjusted TAV patients, whereas we adjusted the diameter for confounders, including age and gender.

The larger prosthesis in BAV compared with TAV is likely related to annulopathy as a part of BAV aortopathy [22, 23]. Furthermore, due to the BAV aortopathy, more additional ascending aorta or aortic arch procedures were also needed. The proximal aorta was repaired in 31% of our BAV patients. Two recent studies reported similar percentages for aortic replacement: 27 and 26% [24, 25]. Since 2002, after a report of a continuing dissection risk during follow-up after isolated AVR in BAV [13], there has been an increasing awareness of possible complications of an unrepaired ascending aorta in BAV. Based on a study published in 2004 [26], the 2006/2007 guidelines recommended replacing the ascending aorta concomitantly when its diameter exceeds 45 mm [27, 28]. However, among the patients in this study who developed an ascending aorta aneurysm requiring surgery, the majority also simultaneously underwent AVR for structural valve deterioration. In these patients it was difficult to retrospectively determine the principle reason for their reoperation [26]. More recent studies reported low incidences of adverse aortic events following isolated AVR in patients with BAV [15, 17, 29]; of these patients, those with BAV *insufficiency* might be a small subgroup with an increased risk [30]. The question may be posed as to whether too many ascending aorta replacements are performed, as the guideline recommendations were based mainly on one publication.

### Limitations

This study is limited by its retrospective design, including the lack of pre-specified criteria for diagnosing BAV at surgery. In our study, 445 out of 894 (50%) patients were excluded for various reasons (died, moved away, operative report missing) (Fig. 1). Our study population is not an ideal sample to study differences in initial patient characteristics. In contrast, it has been established that long-term post-operative survival of BAV patients, also after *isolated* AVR, is not lower than that in TAV patients, provided that the pre-operative diameter of the proximal aorta, the main risk factor for survival, does not exceed 5.0–5.5 cm [15, 16]. Furthermore this is a representative sample of patients with a history of AVS undergoing continued follow-up.

## Conclusion

Hypercholesterolaemia and concomitant CABG were more prevalent in TAV patients at the time of AVS, indicating that an atherosclerotic pathophysiology has a more prominent role in the TAV valvulopathy process, while this is less predominant in BAV patients. These findings suggest that treatment targets should differ in BAV and TAV patients, with more emphasis on statins in the latter. Aortic surgery is performed in almost one third of BAV patients, indicating that future research should focus on whether this high rate of prophylactic surgery is justified.
